# Insights, attitudes, and perceptions about asthma and its treatment: a multinational survey of patients from Europe and Canada

**DOI:** 10.1186/s40413-016-0105-4

**Published:** 2016-05-04

**Authors:** Joaquin Sastre, Leonardo M. Fabbri, David Price, Hans Ulrich Wahn, Jean Bousquet, James E. Fish, Kevin Murphy, Malcolm R. Sears

**Affiliations:** Fundacion Jimenez Diaz, Avenida Reyes Católicos 2, Madrid, 28040 Spain; Department of Oncology Haematology and Respiratory Disease, University of Modena and Reggio Emilia, Via Del Pozzo 71, Modena, I-41124 Italy; Division of Applied Health Sciences, University of Aberdeen, Polwarth Building, Aberdeen, AB25 2ZD UK; Department of Pediatric Pneumology and Immunology, Charité—Universitätsmedizin Berlin, Campus Benjamin Franklin, Augustenburger Platz 1, Berlin, 13353 Germany; Service des Maladies Respiratoires, Hopital Arnaud de Villeneuve, Montpellier-Cedex, 34059 France; Merck & Co., Inc., 1526 Monticello Drive, Gladwyne, PA 19035 USA; Boystown National Research Hospital, 14080 Hospital Road, Boys Town, NE 68010 USA; Firestone Institute for Respiratory Health, McMaster University and St. Joseph’s Healthcare Hamilton, 50 Charlton Avenue East, Hamilton, ON L8N 4A6 Canada

**Keywords:** Asthma, Canada, Controller medications, Europe, Management, Survey, Symptoms

## Abstract

**Background:**

Asthma surveys completed within the past 10 years in the Americas and the Asia-Pacific region have shown significant underassessment of asthma severity in addition to undertreatment of asthma and have suggested the need to improve long-term asthma management. In this study, we examined the frequency of asthma symptoms and severe episodes, patients’ perceived asthma control, and use of asthma medications in Europe and Canada.

**Methods:**

The Asthma Insight and Management survey (54 questions) was conducted in Europe (Germany, Italy, Spain and the United Kingdom) and Canada from June 14 through July 28, 2010. Telephone interviews were conducted with randomly screened patients or parents of adolescents (aged 12–17 years) with asthma; patients younger than 12 years of age were excluded from the survey. Responses were reported separately for each country and in total for all five countries.

**Results:**

Seventy-five thousand three hundered thirty-five households were screened, and 2003 patients were interviewed. The survey respondents represented a wide range of severity. Overall, 26 % of patients reported symptoms daily or most days over the past 4 weeks, but most patients (81 %) perceived their asthma to be well or completely controlled. Over the past year, 41 % of patients had episodes of frequent/severe symptoms, and 50 % reported acute treatment (e.g. hospitalization, emergency visit, unscheduled physician visit) for asthma. Across countries, 52 % of patients reported taking controller medication every day over the past year, 27 % reported not taking any controller medication, and 14 % reported stopping controller treatment for 3 months or longer the last time they stopped. Many patients considered asthma well controlled if each year they had only two urgent doctor visits (50 %), three or four exacerbations (60 %), and/or one emergency room visit (41 %).

**Discussion:**

This is the largest survey of patients with asthma in Europe and Canada in more than a decade.

**Conclusion:**

In 2010, many surveyed patients in Europe and Canada reported features indicating uncontrolled asthma, yet the majority believed they were well controlled, indicating that they had low expectations of long-term asthma management. Use of controller medications was substantially less than recommended in treatment guidelines.

**Electronic supplementary material:**

The online version of this article (doi:10.1186/s40413-016-0105-4) contains supplementary material, which is available to authorized users.

## Background

Asthma is a global public health problem affecting an estimated 235 million people [1]. Poorly controlled asthma results in symptoms that are burdensome and that may precede clinically significant exacerbations. The National Review of Asthma Deaths (NRAD) reported that asthma mortality in the United Kingdom was among the highest in Europe and reviewed information on 195 patients who died from asthma between February 2012 and January 2013 [2]. Only 23 % of these patients had been provided personal asthma action plans to identify factors that trigger or exacerbate asthma. The NRAD also found evidence that reliever medication was overused and preventer medication was underused in the United Kingdom [2]. Patient surveys reported in the previous two decades have indicated an inadequate level of asthma control based on symptoms, activity limitations and acute treatment (e.g. hospitalization) [3–13]. The 1996 European Community Respiratory Health Survey (ECRHS) found a high rate of asthma attacks and asthma medication use among asthmatic adults aged 20 to 44 years to be particularly high in western Europe, the United Kingdom, Sweden, Australia, New Zealand and the United States; this contrasts with particularly low rates in Estonia, Greece and Spain [3]. A survey using the same sampling strategy and instruments as the ECRHS was conducted in 1993 to 1994 at six sites across Canada, and it evaluated the sex-specific prevalence of asthma attacks and asthma medication use. This survey found the prevalence of asthma attacks and asthma medication use to be higher among women than men in all sites, except for a higher rate of asthma attacks among men in Vancouver [4]. The International Study of Asthma and Allergies in Childhood (ISAAC; 1998) reported asthma-symptom prevalence rates for 13- to 14-year-old children in 56 countries that ranged from 1.6 % (Indonesia) to 36.8 % (UK) In addition, up to approximately 30 % of Canadian children in the ISAAC survey reported asthma symptoms over the past 12 months [5]. In the 1999 Asthma Insights and Reality in Europe (AIRE) study of adults and children with asthma (*N* = 2803) in seven European countries, only 5 % of patients met all of the criteria for asthma control, and 7 % of patients had overnight hospitalization in the previous year [6]. A later analysis of AIRE study data found overall that >50 % of children and >40 % of adults with severe asthma perceived their asthma to be well controlled and that disparities between patient perceptions and actual control were substantial although variable among the seven countries [7]. In a 1996 telephone survey of Canadian patients >16 years of age with physician-diagnosed asthma (*N* = 829), 43 % reported frequent or very frequent symptoms of asthma. Furthermore, 26 % reported use of urgent care services in the past year, and 21 % reported an asthma flare-up (i.e. exacerbation) or need for urgent treatment within the past 6 months [14]. Additional survey results have corroborated a lack of control considered adequate at the time by expert practitioners [10, 15].

The main goal of this Europe and Canada (EUCAN) Asthma Insight and Management (AIM) survey was to re-examine patient-reported beliefs concerning their asthma and its management and to analyze whether these beliefs have changed since previous surveys. In particular, the purpose of the study was to explore and document asthma-related patient perceptions, behaviors, and presentation patterns. Another goal of this survey was to gain a better understanding of the tools and techniques physicians currently use to assess and manage their patients’ asthma and to gauge the extent to which physicians are following Global Initiative for Asthma (GINA) guidelines [16] with regard to asthma assessment and management techniques.

## Methods

### Study design

#### Patients

The EUCAN AIM survey was conducted in Germany, Italy, Spain, the United Kingdom, France, and Canada from June 14 through July 28, 2010. All questions were originally drafted in English and translated to local languages. The translated questions were back-translated to English and compared for meaning against the original English version. Findings from France are not included here owing to inaccurate translation of the questionnaire, which was identified before completion of the survey in France. Households were sampled by random-digit dialing and systematically screened to determine whether there were any household members with current physician-diagnosed asthma, and if so, whether they (or their child) had used any prescription medicine for control, prevention, maintenance, or regular treatment of asthma in the past year.

This survey was restricted to adults and adolescents with current asthma, so households with no current patients with asthma or only patients younger than 12 years with pediatric asthma screened out of the survey. Patient consent was obtained before the interview began, and the respondent could refuse to cooperate at any point in the interview.

#### Outcomes

The survey was developed by the public opinion research organization Abt SRBI, Inc. (New York, NY, USA) with input from the steering committee and from prior published AIM surveys. Telephone interviews lasting approximately 40 min were conducted with patients or parents of adolescents (aged 12–17 years) with asthma. Interviewers with survey organizations based in each target country read a standard text to introduce the study and ask the survey questions. The data were not collected by doctors or other health professionals.

All patients had physician-diagnosed asthma and used asthma medication or had asthma symptoms in the past year. The survey had 54 questions (Additional file 1), and responses were reported separately for each country as well as total estimates for all five countries.

Understanding of asthma exacerbations was explored using several terms (asthma flare-ups, asthma exacerbations, asthma attacks, and asthma worsening). The frequency and duration of exacerbations or asthma worsening was documented. Respondents were provided several scenarios of exacerbations and urgent doctor visits and asked whether these indicated well-controlled asthma. Details of asthma assessment and management by the patient and their healthcare provider, use of oral corticosteroids, frequency of use of a reliever inhaler, and use of controller medications were ascertained.

#### Sample size and statistical analysis

The maximum margin of error for a sample size of 400 (individual country samples) is ±4.9 percentage points at the 95 % confidence level. Descriptive comparison of response rates among countries is provided, but no statistical analysis of the results was performed.

## Results

### Demographic characteristic of patients

A total of 75,335 households were screened, and 2003 patients (400–403 per country) were interviewed (Tables 1, 2). Data are not available for the number of qualified patients with asthma who were not interviewed. The interviewed patients were adults or parents of adolescents (age 12–17 years) with physician-diagnosed asthma who had, in the previous year, taken asthma medication or had experienced asthma symptoms. The proportion of females ranged from 64 % in the United Kingdom to 69 % in Italy, overall mean 66 %. The mean age of respondents ranged from 42 years in Germany to 51 years in the United Kingdom, overall mean 47 years. The respondents’ mean age at diagnosis of asthma was 25 years, with the mean age at asthma diagnosis ranging from 22 years in Canada to 27 years in Germany (Tables 1, 2).

**Table 1:** Study design

Population	Sampling frame	Interview length
Adults and parents of adolescents (age 12–17 years) with asthma (physician diagnosed and past year medication or asthma symptoms)	Telephone screening of national random digit dialing sample of households	Mean: 37.9 min

### Main results

**Fig. 1:**
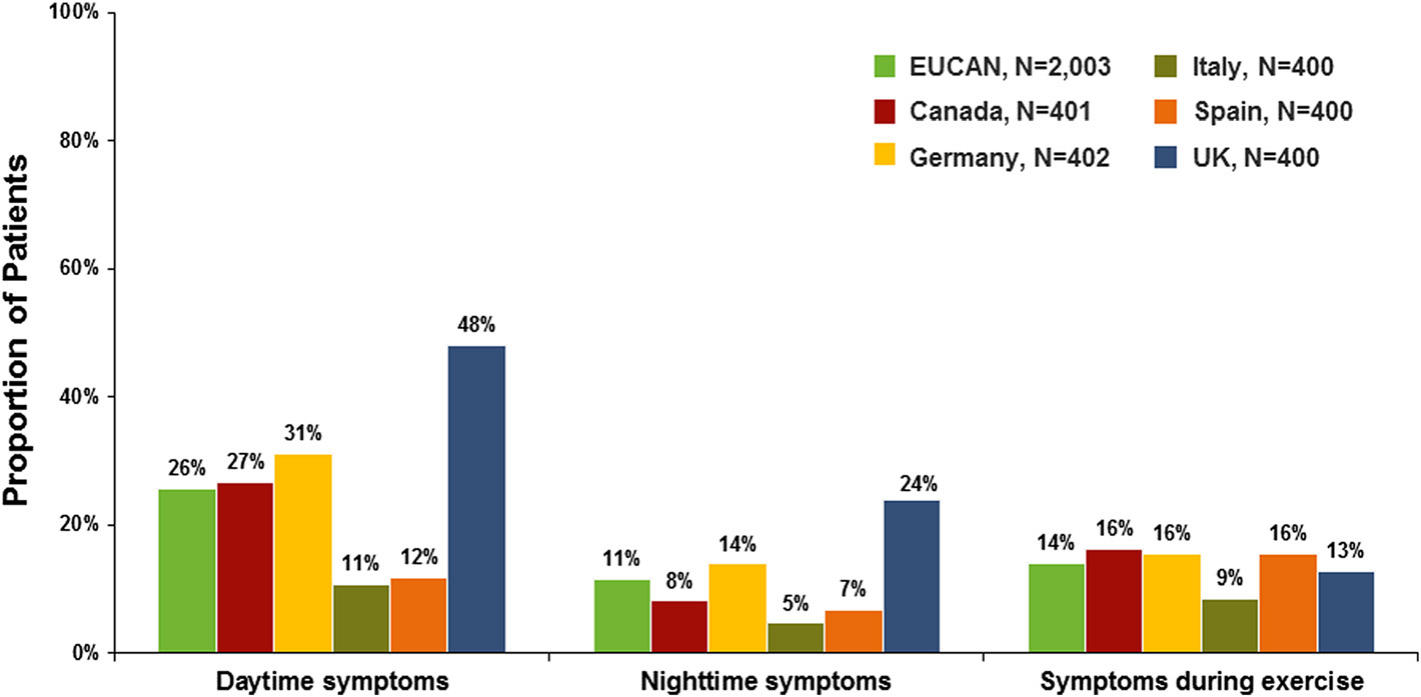
Patient-reported frequency of symptoms in the past 4 weeks (every day or most days)

On average, patients reported that they had symptoms for 3 years before their asthma diagnosis, although subjects answering the questionnaire represented a wide range of severity. Overall, more than one in four patients reported symptoms every day or most days over the past 4 weeks. In addition, 11 % of patients reported symptoms every night or most nights over the past 4 weeks, and 21 % of patients reported symptoms during exercise, play, or physical exertion every day or most days over the past 4 weeks (Fig. 1). While more than 25 % of patients reported that daytime symptoms occurred every day or most days, indicating moderate or severe persistent asthma, over half (56 %) of patients reported that daytime symptoms occurred less than once a week, which indicates they had mild or intermittent asthma. The majority of patients (61 %) reported allergens (ie., dust, pollen, grass, animals, and insects) to be the most common factor to trigger their asthma or make their symptoms worse. In addition, 31 % of patients reported environmental factors (i. e., pollution, chemicals, fumes, tobacco smoke, and perfume) and 28 % reported weather changes as asthma triggers. Other asthma triggers reported less frequently were exercise (21 %), emotional factors (11 %) and viruses/colds (11 %).

**Fig. 2:**
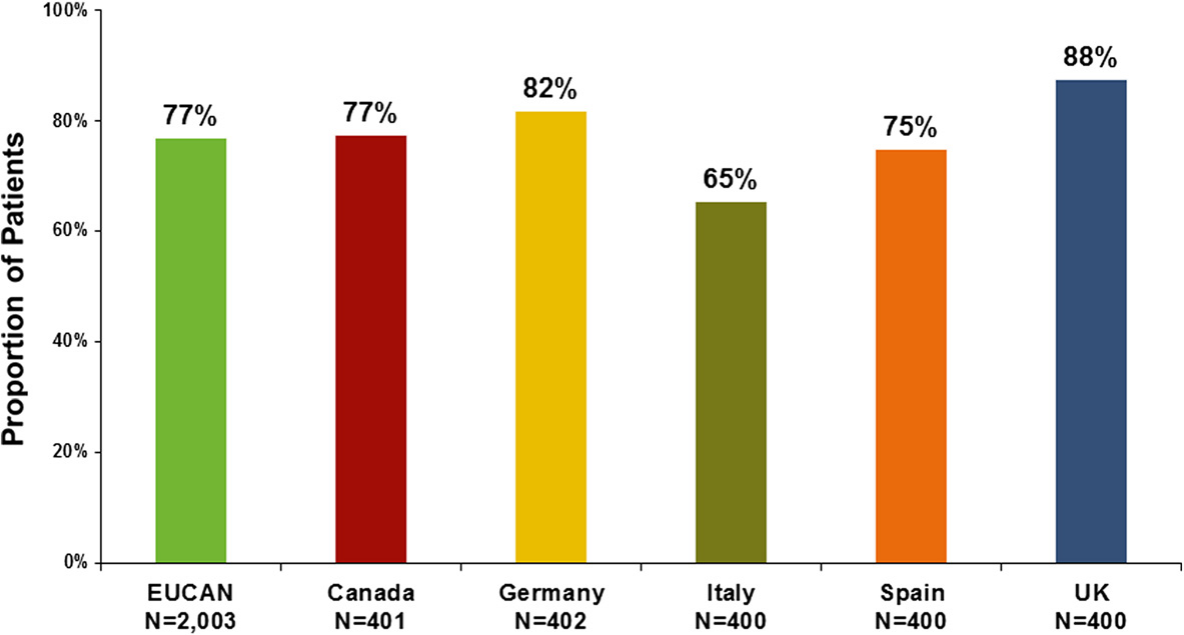
Proportion of patients who perceived their asthma as completely or well controlled in the past 4 weeks

Despite the relatively high proportion of asthma patients reporting daytime symptoms, nighttime symptoms, and symptoms during exercise or exertion on every day or most days during the past 4 weeks, 81 % of patients perceived their asthma to be well or completely controlled over this period (Fig. 2). Using objective criteria based on GINA guidelines, asthma control in the past 4 weeks was worse than the perceived asthma control reported by patients. Only 18 % would be classified as having controlled asthma according to the GINA guidelines, while 58 % would be classified as partially controlled, and 24 % as uncontrolled.

### Asthma burden

**Fig. 3:**
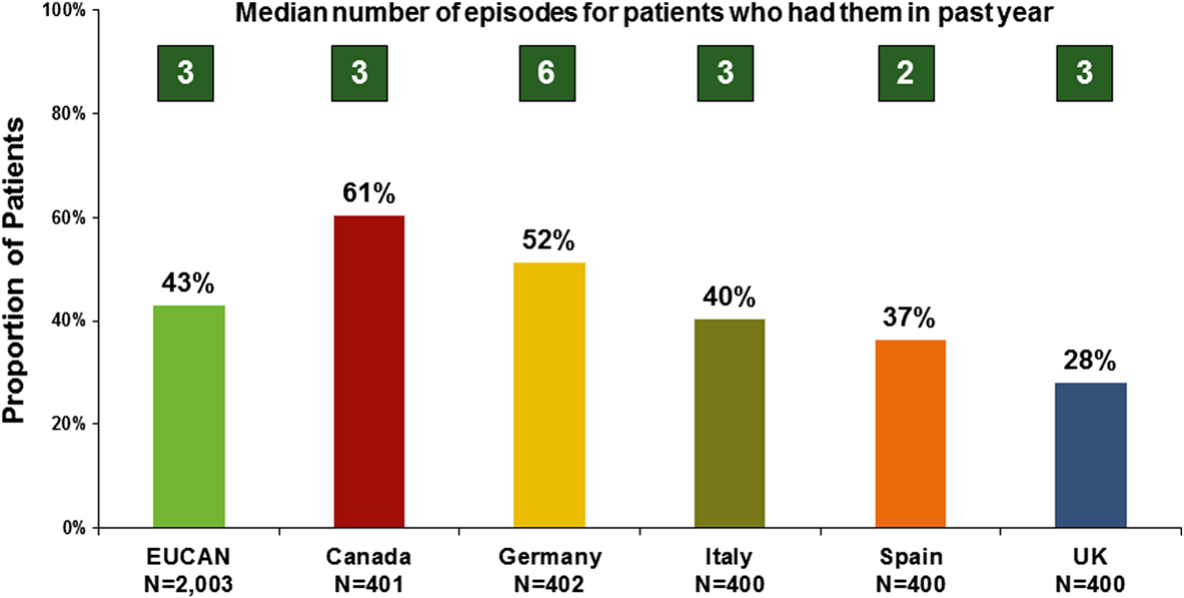
Proportion of patients reporting episodes in the past 12 months when asthma symptoms were more frequent or more severe than normal

Overall, nearly half of adults and adolescents with asthma in Europe and Canada reported that asthma symptoms are extremely or moderately bothersome when they have them. Furthermore, approximately two of five survey respondents reported episodes when asthma symptoms were more frequent or more severe than normal in the past 12 months. However, there was substantial variation across countries in the proportion of asthma sufferers who reported these episodes of symptom worsening (more frequent or more severe) in the past 12 months. The proportion of patients reporting symptom-worsening episodes in the past 12 months ranged from 28 to 61 % (Fig. 3).

**Fig. 4:**
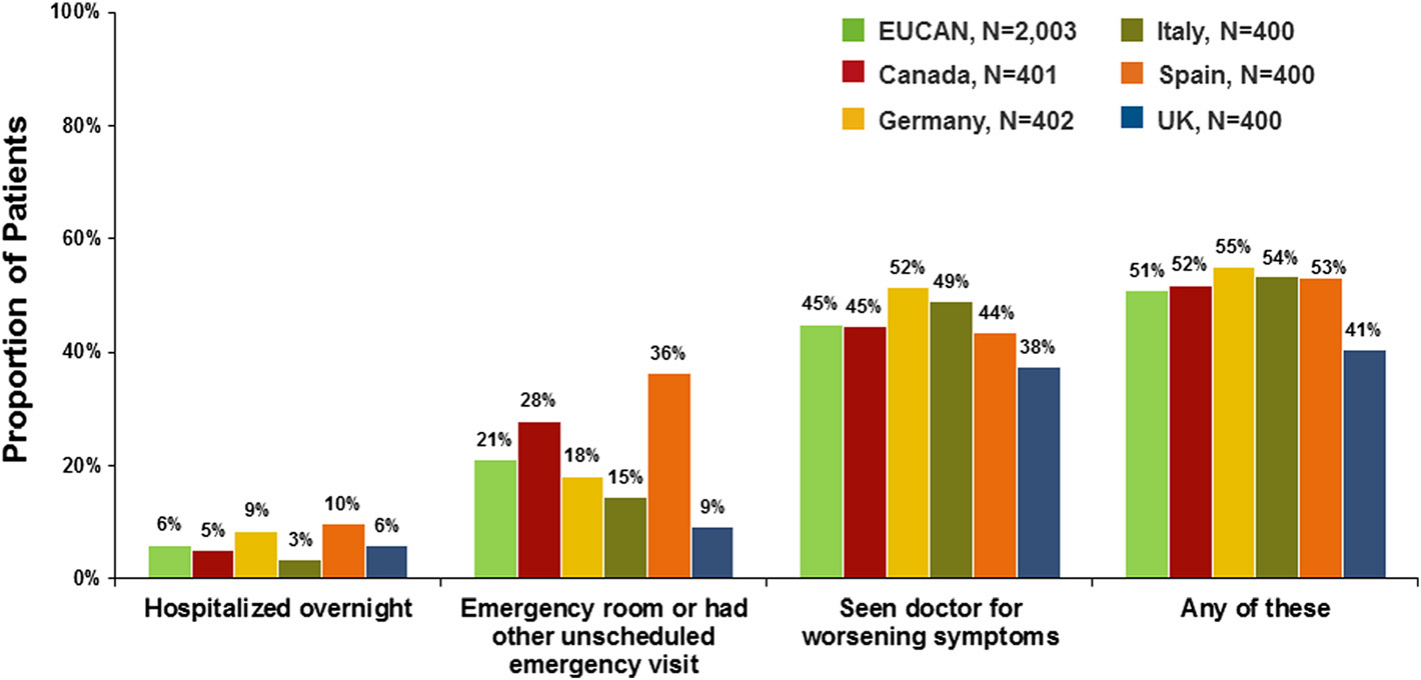
Proportion of patients reporting acute treatment for asthma in the past year

Overall, patients with asthma in Europe and Canada reported a median of three asthma exacerbations in the past 12 months. The mean duration of these episodes was 12 days: 8 days in Canada, 11 days in the UK, 13 days in Germany, 14 days in Spain, and 15 days in Italy. The median duration of these episodes was 3 days in all countries except Spain, where the median duration was 5 days. It is likely that exacerbations of long duration occurred among many patients in each country to push the means so far above the medians. Although the particular pattern of acute care for asthma varies among countries depending on their healthcare system, half of the adults and adolescents with asthma in Europe and Canada reported acute treatment (e.g. hospitalization, emergency visit, unscheduled physician visit) for asthma in the past year (Fig. 4). A substantial portion of the asthma patient population experience what it perceives as life-threatening asthma episodes. Asthma exacerbations are physically threatening and emotionally significant for patients. One-third or more of all adults and adolescents with asthma reported having had an asthma episode so bad they thought their lives were in danger (Table 3). The survey questions did not allow discernment of the severity of exacerbations among the respondents. However, it can be presumed that up to 51 % of patients, who reported acute treatment for asthma in the past year, had severe exacerbations.

**Table 2:** Study design

Country	Households screened, n	Asthmatics interviewed, n	Female, %	Mean age, years	Mean age at Diagnosis, years
Canada	7405	401	67	45	22
Germany	22,293	402	67	42	27
Italy	13,620	400	69	50	25
Spain	24,404	400	65	44	25
United Kingdom	7613	400	64	51	25
TOTAL	75,335	2003	66	47	25

In addition to the reported occurrence of frequent/severe asthma episodes and exacerbations, the EUCAN AIM survey demonstrates the significant impact of asthma on patients’ day-to-day lives. Overall, 19 % of adults and adolescents reported that their asthma caused them to miss work or school in the past year, with a median of 7 days missed (Table 3), 31 % of patients reported that they had episodes of sufficient severity to be short of breath while sitting still, 23 % reported that they had severe episodes limiting their speech to only a few words at a time, and 28 % reported that they had been awakened frequently at night by asthma symptoms.

### Asthma treatment

More than half of patients reported that they had taken prescription controller medicines for their asthma in the past 4 weeks. The proportion of patients with asthma using prescription controller medicines in the past 4 weeks was 49 % in Italy, 58 % in the United Kingdom, 59 % in Canada, 60 % in Spain, and 61 % in Germany.

**Fig. 5:**
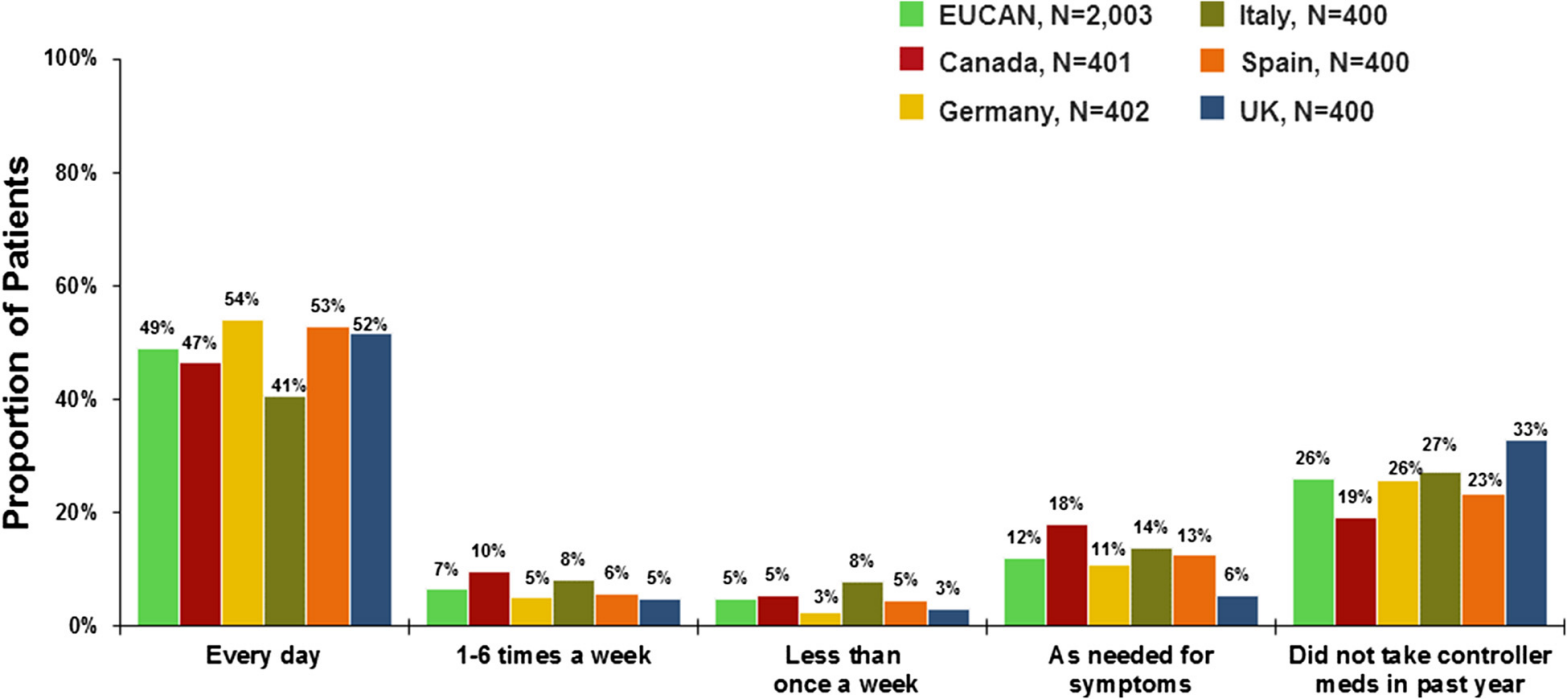
Patient-reported frequency of asthma controller medicine use in the past year

**Fig. 6:**
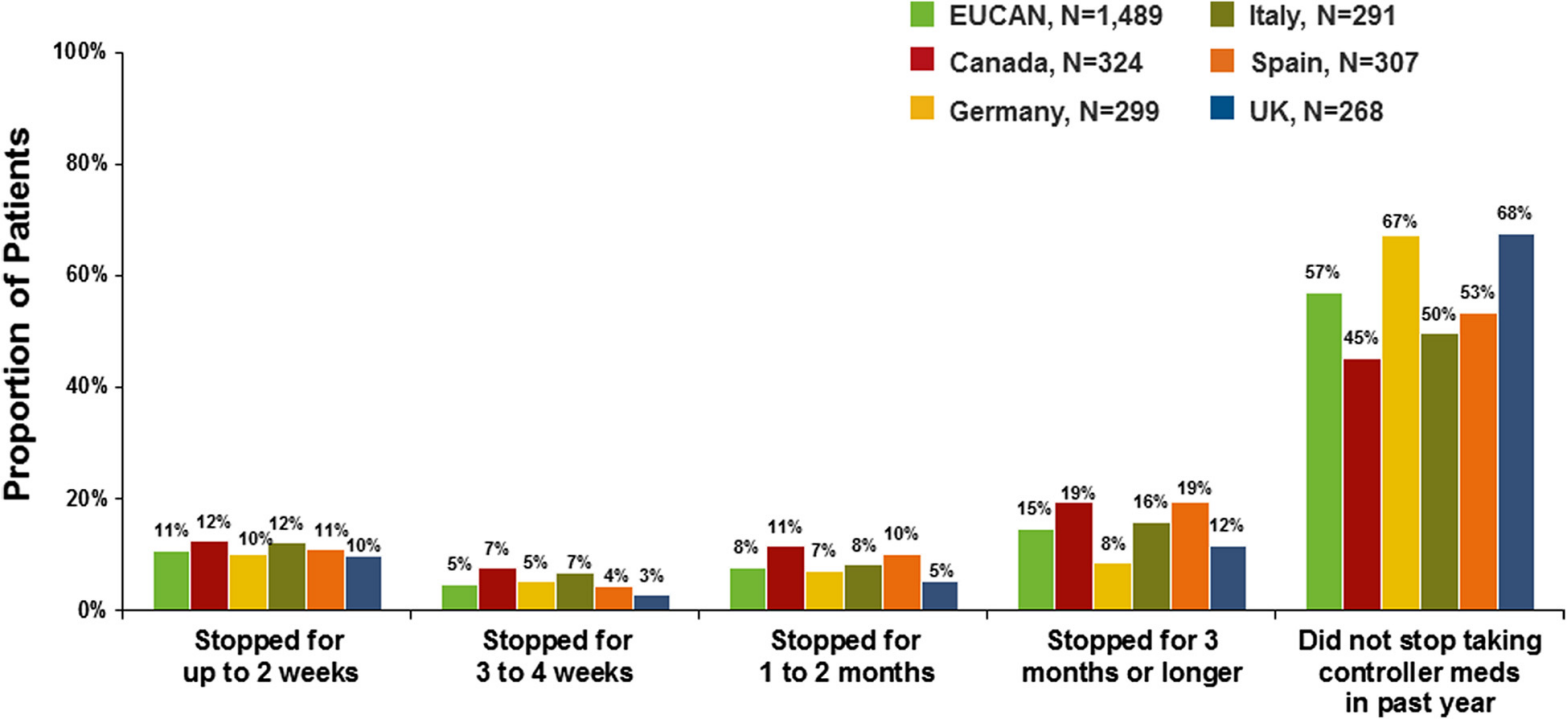
Proportion of patients who stopped taking asthma controller medicine in the past year (as percentage of those taking controller medicine at any time of the year)

Use of controller medications reported in the EUCAN AIM survey shows disagreement between guideline recommendations for daily use and patient choices. Overall, more than half of patients reported taking controller medication every day over the past year. However, approximately one-quarter of subjects reported not taking any controller medication in the past year (Fig. 5). Correlations between usage of medication and reported asthma control indicate that some patients who reported infrequent symptoms also reported use of daily controller therapy (Table 4), a treatment that patients with more severe asthma would require. Among patients who used prescription controller medication at any time during the past year, up to 12 % of patients reported stopping controller treatment for at least 1 to 2 weeks the last time they stopped (Fig. 6).

**Table 3:** EUCAN AIM survey responses

Survey questions	EUCAN	Canada	Germany	Italy	Spain	UK
	(*N* = 2003)	(*n* = 401)	(*n* = 402)	(*n* = 400)	(*n* = 400)	(*n* = 400)
Control classification based on 2009 GINA, %						
▪ Well controlled	18	16	10	17	12	24
▪ Partly controlled	58	59	66	64	64	51
▪ Uncontrolled	24	25	24	20	24	25
Had episodes in the past 12 months when asthma symptoms were more frequent or more severe than normal, %	38	61	52	40	37	28
▪ Median number of episodes, n	3	3	6	3	2	3
Have ever had an asthma episode perceived as life threatening, %	33	40	39	32	44	27
Have in the past year had an asthma episode perceived as life threatening, %	9	10	14	10	13	5
Missed work or school in the past year due to asthma, %	19	21	24	16	23	12
▪ Median number of days missed, n	7	6	10	10	5	4
Used quick relief medication for asthma in past 4 weeks, %	68	69	59	66	71	80
Used inhaler for quick relief of asthma symptoms at least once a week over the past year, %	46	43	35	34	49	56
Used an oral steroid (pill or liquid) to manage asthma symptoms in the past year, %	32	30	41	50	39	21
▪ Median number of times an oral steroid was used for 3 days or longer in the past year, n	2	3	2	3	2	2
How the doctor usually assesses asthma, %						
▪ Has the patient fill out a questionnaire	7	7	13	2	6	6
▪ Gives the patient a breathing test or spirometry	47	24	54	25	59	50
Has a doctor-developed written action plan for asthma treatment, %	23	18	25	39	45	15
Agree with the following statements, %						
▪ Maintenance medicines should be taken every day	66	64	65	57	64	69
▪ Maintenance medicines are not necessary when asthma symptoms are not experienced regularly	48	59	60	48	59	39
▪ Rescue medicines can be used every day if needed	67	75	58	59	62	75
▪ Fear of asthma exacerbations keeps me from doing the things I want to	26	25	32	35	36	18
▪ I worry about using oral steroids, like prednisone	34	54	32	30	40	34

Nearly seven of 10 patients with asthma in Europe and Canada reported that they had taken prescription quick-relief or rescue medicines for their asthma in the past 4 weeks (Table 3). This means that about 30 % had not used an inhaler in the last 4 weeks, emphasizing that many had mild or intermittent disease where one would not expect regular controller treatment to be mandatory. Forty-six percent of all subjects (range 34 % in Italy to 56 % in UK) had used an inhaler for quick relief of asthma symptoms at least once a week over the last year, indicating the presence of persistent asthma. Among patients who reported having symptoms every day, 23 % did not use rescue medications in the past year, although 66 % of these patients used controller medication every day (Table 4). Overall, however, at least 19 % of patients with daily symptoms had not used any inhaled medication in the past year. The proportion of EUCAN AIM respondents who reported taking an oral steroid (pill or liquid) to manage their asthma symptoms in the past 12 months ranged from 21 % in the United Kingdom to 50 % in Italy. On average, patients took oral steroids for 3 days or longer twice in the past year (Table 3).

In a further analysis, 19 % of patients who experienced symptoms every day did not take a controller medicine in the past year, and 11 % took it less than once a week. In contrast, among patients who experienced symptoms less frequently than once a week, 33 % did not take any controller medication in the past year (Table 4).

### Attitudes about asthma

**Fig. 7:**
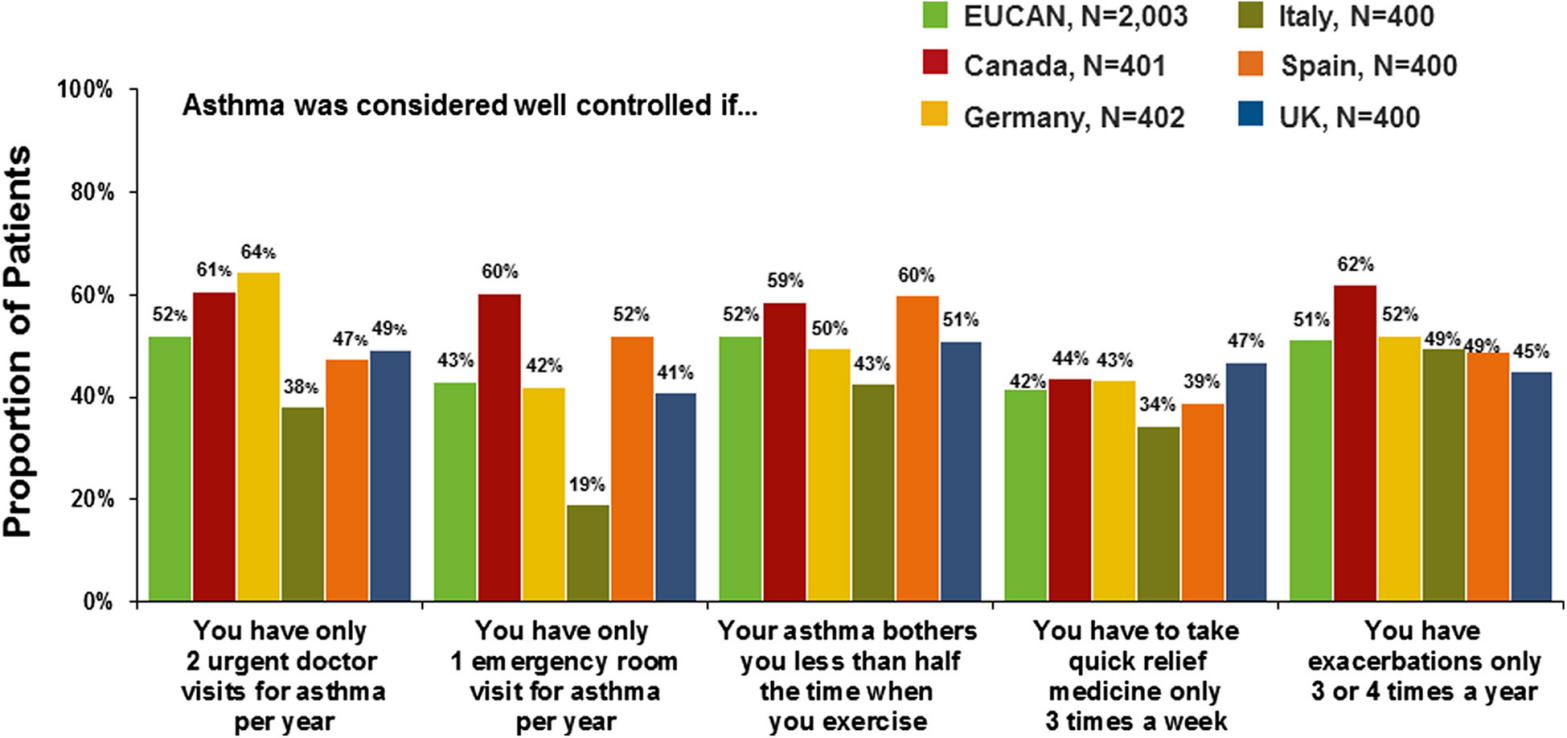
Patient-reported attitudes about asthma management and control

Patients with asthma in EUCAN had low expectations of disease management. At least half of patients considered their asthma well managed if their asthma bothers them less than half the time when they exercise, well managed if they only have two urgent care visits for asthma per year, and well controlled if they have only three or four exacerbations a year (Fig. 7). In Europe and Canada, more than one in four patients with asthma agree that fear of asthma exacerbations keeps them from doing the things that they want to do (Table 3). About one-third of asthma patients in the European countries also agree that they worry about using oral steroids. Concerns about the use of oral steroids are much higher in Canada, where more than half of asthma patients worry about using oral steroids.

When survey respondents were asked how their doctor usually assesses asthma, 2 to 13 % of asthma patients reported that their doctor had them fill out a questionnaire. About one-quarter of patients with asthma in Canada and Italy, as well as at least half of asthma patients in the United Kingdom, Germany, and Spain said that their doctor usually assessed their asthma with a breathing test or spirometry. Many patients may have simply had peak flow monitoring rather than full spirometry. The proportion of patients reporting that they had a doctor-developed written action plan for asthma treatment was one-quarter or less in Germany, Canada, and the United Kingdom, in contrast to over one-third in Italy and Spain.

Overall, two-thirds of patients with asthma agreed that maintenance medicines should be taken every day. However, more than half of patients in Spain, Canada, and Germany also agree that maintenance medicines are not necessary when asthma symptoms are not experienced regularly. Another negative finding is that two-thirds of patients with asthma agreed with the statement that rescue medicines can be used every day if needed. Moreover, about one-third of patients with asthma in the European countries reported that they worry about using oral steroids. Patients’ attitudes and beliefs about treatment are important because they may affect asthma management and control.

## Discussion

The EUCAN AIM is the largest survey of patients with asthma in Europe and Canada in more than a decade. The survey was conducted by telephone interview (median time, 39.7 min) in 2420 adults or parents/guardians of adolescents (aged 12–17 years) with current diagnosed asthma. Based on national probability samples of approximately 400 persons aged 12 years and older with current asthma, the study showed significant unmet needs in asthma care in Europe and Canada, with persistence of significant asthma symptoms and exacerbations, low expectations, and marked undertreatment. Up to 19 % of those taking controller medication reported gaps in treatment of 3 months or longer. The study is limited, however, in that between-country results were not analyzed statistically and self-selection bias may preclude the generalizability of study findings to the general population. Another study limitation is that thorough data on correlations between asthma severity and medication usage were not obtained in the survey. About half of patients were very infrequent users of reliever treatment, which means almost certainly that they had mild or intermittent disease and were unlikely to be prescribed maintenance medication. The finding of low reliever use among some patients suggests they had very mild disease or were among those who reported using controller medications every day and so did not feel the need for rescue medication. However, nearly one in four patients reported that they used rescue medication every day but had daytime symptoms less than once a week. Given that assessment of asthma severity determines the medications that are required, it is not reasonable to anticipate that patients with different degrees of asthma severity would require the same controller or rescue therapy.

Patients in this survey experienced relatively frequent asthma symptoms and approximately 40 % reported symptoms to be moderately to extremely bothersome. Some patients with troublesome asthma who reported frequent bronchodilator use were not prescribed controller therapy. Findings from the National Review of Asthma Deaths (NRAD) indicate that 14 % (*n* = 27) patients who died from asthma in the United Kingdom were prescribed a single-component quick-relief medication around the time of death. At least 3 % (*n* = 5) patients were on reliever monotherapy without controller treatment [2]. In EUCAN AIM, nearly half of patients who reported episodes of symptom worsening also reported that they needed hospitalization, emergency room care, or other unscheduled medical visits for asthma episodes or exacerbations over the past year. Over the past year, nearly one-third of patients had required a course of oral steroids, and nearly 10 % of patients reported having an episode as being sufficiently grave that they felt their life was in danger.

While our findings showed similarities across countries in patient-perceived level of asthma control, they also revealed that asthma is far from being well controlled. Our survey revealed a huge discrepancy between the proportion of asthma patients agreeing with the statement that their asthma is well or completely controlled (80 %) and the proportion who would be classified as having controlled asthma according to GINA guidelines (18 %) [16]. The patients with asthma who were surveyed had low expectations about what constitutes well-controlled asthma given their reported symptom frequency, exacerbations, use of quick-relief medicine, and emergency room visits or other urgent care. This indicates that patients are satisfied with what clinicians consider to be an unacceptable burden of disease.

The untoward consequences of uncontrolled asthma and its impact on costs and exacerbation risk have been reported in several epidemiologic studies [17–19]. In addition, the burden of asthma identified in previous surveys of the region [4, 6–8, 14] continues to exist, even though effective controller medications are available and treatment guidelines have been widely disseminated.

Patient-reported activity limitations, missed school/work days, number of emergency visits, and number of hospitalizations in the present survey suggest a lack of improvements in asthma control in Europe and Canada over the past decade, and reveal significant unmet needs with regard to the current state of asthma care in these countries. Although most survey respondents recognized that maintenance medication for asthma should be used every day, many reported that they did not take prescription controller medicines daily. Other patients discontinued controller medicines altogether for a week or longer in the past year, and still other asthma patients had not taken asthma maintenance medicines in the past year. These findings are consistent with parallel AIM surveys conducted in the United States [20, 21], the Asia-Pacific region [22], and Latin America [23].

Similarities and differences exist between EUCAN and US AIM survey results (Table 5). In addition, the 1999 AIRE study, which surveyed 2803 patients with asthma in France, Germany, Italy, the Netherlands, Spain, Sweden, and the United Kingdom found that 38 % of children and 50 % of adults had daytime symptoms at least once a week. The EUCAN AIM survey finding that 26 % of patients reported daytime symptoms every day or most days suggests that patients’ control of their asthma symptoms has not improved in the past decade despite published guidelines and availability of effective controller treatments. Furthermore, the AIRE and EUCAN AIM surveys showed similar discrepancies between patient-reported asthma control and control levels based on GINA guidelines.

**Table 4:** Daytime symptom frequency and use of rescue and controller medication among EUCAN patients

Frequency of daytime symptoms	Frequency of rescue medication use, n (%)^a^					Frequency of controller medication use, n (%)^a^			
	Every day	3–6/week	1–2/week	<1/week	Not used in past year	Every day	1–6 / week	<1/week	Not used in past year
Every day	159 (52)	23 (8)	18 (6)	33 (11)	70 (23)	199 (66)	12 (4)	32 (11)	59 (19)
(*n* = 303,^a^ 288^b^)									
Most days	106 (47)	24 (10)	15 (7)	35 (15)	48 (21)	149 (66)	18 (8)	30 (13)	31 (13)
(*n* = 226,^a^ 213^b^)									
Twice/week	66 (27)	26 (11)	30 (12)	44 (18)	74 (31)	116 (49)	31 (13)	40 (17)	52 (22)
(*n* = 240,^a^ 248^b^)									
Once/week	32 (24)	11 (8)	16 (12)	35 (26)	38 (29)	62 (47)	10 (7)	26 (20)	35 (26)
(*n* = 132,^a^ 133^b^)									
Less than once/week	272 (23)	57 (5)	93 (8)	332 (29)	407 (35)	507 (44)	58 (5)	210 (18)	386 (33)
(*n* = 1162,^a^ 1120^b^)									
Total	635 (31)	140 (7)	171 (8)	478 (23)	638 (31)	1033 (50)	129 (6)	337 (16)	562 (27)
(*n* = 2063,^a^ 2002^b^)									

^a^Sample sizes and proportions weighted by the size of the population and the prevalence of asthma in each of the countries

^b^Unweighted sample sizes

**Table 5:** Similarities and differences between EUCAN and US AIM survey results

	Proportions of patients (%)	
Patient reports	EUCAN AIM	US AIM [20, 21]
Had well-controlled asthma based on guidelines	18^a^	29
Had severe asthma episodes in the past year	38^b^	52
Ever had an asthma exacerbation perceived as life-threatening	33	36
Missed work or school in the past year due to asthma	19	22
Had overnight hospitalization for asthma in the past year	7	6
Agreed with the statement that:		
• maintenance medication should be taken every day	66	74
• maintenance medication is not necessary when asthma symptoms are not experienced regularly	48^c^	40
• rescue medication can be used every day if needed^d^	67	67
Used prescribed controller medication in the past 4 weeks	57	70
Used quick-relief inhaled medication at least once a week over the past year	43	51
Used an oral steroid (pill or liquid) to manage asthma symptoms in the past year	32	35
Worry about using oral steroids, like prednisone	34^e^	≥52^f^
Had a doctor-developed written action plan for asthma treatment	23^g^	32^h^

Some of the country-specific EUCAN results were similar to the US results

^a^in the United Kingdom, 25 % of patients reported that they had well-controlled asthma

^b^in Germany, 52 % of patients reported that they had severe asthma episodes over the past year, with a median of 6 severe episodes during the year (the median was 3 in all other countries, including the US)

^c^in the United Kingdom, 39 % of patients agreed with the statement that maintenance medication is not necessary when asthma symptoms are not experienced regularly; this finding reveals misalignment of patient beliefs and asthma management guidelines

^d^patients who need to use rescue medication every day are likely to have poorly controlled or severe asthma

^e^in Canada, 54 % of patients reported that they worry about using oral steroids

^f^patients’ concern about oral steroid use was stratified by their level of asthma control (well controlled [52 %], not well controlled [56 %], or very poorly controlled [61 %])

^g^in Italy and Spain, 39 and 45 % of patients, respectively, reported that they had a doctor-developed written action plan for asthma

^h^only 25 % of US patients had ever completed the Asthma Control Test [25]

The reporting of regular, as needed quick-relief inhaler use by two-thirds of patients in the EUCAN AIM survey is consistent with the findings of a survey involving 1022 patients with asthma in five European countries, in which 64 % of patients reported that treatment with immediate results (ie, quick relief) gave them a reason for regular use of that treatment [24].

## Conclusions

In 2010, many patients in Europe and Canada had low expectations of long-term asthma management and unacceptable levels of asthma control, despite the availability of effective medications. Furthermore, their reported use of controller medications did not reflect guideline recommendations for uninterrupted daily use. Although more than half of those surveyed reporting taking controller medication every day, discontinuation of this medication was common. The survey findings yield a number of important insights about asthma and the current state of asthma management. Many patients believe that their symptoms are well controlled, despite reporting substantial symptoms and morbidity. Patients with asthma have very low expectations for controlling their disease, with 60 % considering their asthma to be well controlled if they have only three or four exacerbations a year.

Patient acceptance of asthma burden, as well as the apparent lack of conviction on treatment recommendations and goals, are persistent problems which need to be addressed by improved implementation of asthma guidelines and patient education.

## Additional file

Additional file 1: EUCAN AIM Survey Questions. (PDF 108 kb)
