# Supplementary material for: Insights, attitudes, and perceptions about asthma and its treatment: a multinational survey of patients from Europe and Canada
Source: World Allergy Organ J. 2016 May 4;9:13. doi: 10.1186/s40413-016-0105-4 (PMC4855503; doi:10.1186/s40413-016-0105-4)
Supplement: Additional file 1: — EUCAN AIM Survey Questions. (PDF 108 kb) [file 40413_2016_105_MOESM1_ESM.pdf]

EUCAN AIM Survey Questions

The Base survey population was all respondents, unless noted otherwise.

Asthma symptoms, frequencies and patterns

1. How long did (you/your child) have symptoms before a diagnosis of asthma was made?

2. What things usually trigger or make (your/your child's) asthma symptoms worse?

3. In the past 4 weeks, (have you/has your child) had a cough, or wheezing, or shortness of breath, or chest tightness during exercise, play, or physical exertion?

4. In the past 4 weeks, (have you/has your child) been awakened by a cough, or wheezing, or shortness of breath, or chest tightness during the night?

5. In the past 4 weeks, (have you/has your child) had a cough, or wheezing, or shortness of breath, or chest tightness during exercise, play, or physical exertion?

6. How often did (you/your child) have these symptoms during the day: every day, most days, at least twice a week, once a week, at least twice a month, or once a month?

7. How often did (you/your child) have these symptoms at night: every night, most nights, at least twice a week, once a week, at least twice a month, or once a month?

8. How often did (you/your child) have these symptoms during exercise, play, or physical exertion: every day, most days, at least twice a week, once a week, at least twice a month, or once a month?

9. Overall, how well would you say that (your/your child's) asthma has been controlled in the past 4 weeks? Would you say it was completely controlled, well controlled, somewhat controlled, poorly controlled, or not controlled at all?

10. Would you describe (your/your child's) asthma as seasonal or does it occur throughout the year?

11. During what months of the year are (your/your child's) asthma symptoms more frequent or more severe than normal?

    Base: Experienced episode at particular time of year

12. During the worst month in the past 12 months, did (you/your child) have:
    - Coughing during the day?
    - Coughing, wheezing, etc during exercise?
    - Shortness of breath during the day?
    - Wheezing or whistling in chest?
    - Coughing up phlegm?
    - Chest tightness?
    - Awakened at night with coughing?
    - Awakened at night by shortness of breath?

    every day, most days, at least twice a week, at least twice a month, less than that, or never?

13. When (you have/your child has) them, how bothersome are the following symptoms? Is (ITEM) usually extremely bothersome, moderately bothersome, slightly bothersome, or not bothersome?

14. Were there any episodes in the last 12 months when (your/your child's) asthma symptoms were more frequent or more severe than normal?

15. How many different episodes in the past 12 months were (your/your child's) asthma symptoms more frequent or more severe than normal?

    Base: Had severe episode in past year

16. On average, how many days did those episodes last (when your/your child's asthma symptoms were more frequent or more severe)?

    Base: Had severe episode in past year

17. During the past 12 months, (have you/has your child) had an asthma episode when:
    - Even when you are sitting still, you felt short of breath?
    - You could only say a few words at a time because you were so short of breath?
    - You were awakened at night by your asthma symptoms frequently?

Patients' experiences with asthma exacerbation

18. (Have you/Has your child) been hospitalized overnight for asthma in the past 12 months?

19. (Have you/Has your child) gone to a hospital emergency room, or walk-in clinic (CA)/gone to an accident and casualty room/been visited by an emergency mobile doctor or gone to an emergency room (GER)/gone to an emergency room, or been visited by a night-shift doctor (ITA)/gone to an emergency room (SPN)/gone to an accident and casualty room or after-hours service (UK) for asthma symptoms in the past 12 months?

20. Has (your/your child's) asthma caused any other unscheduled emergency visits to a doctor's office, clinic, or somewhere else in the past 12 months?

21. In the past 12 months, (have you/has your child) seen a doctor or other health-care provider because of asthma exacerbations or worsening symptoms?

22. How many times (have you/has your child) been hospitalized for asthma in the past 12 months?

    Base: Hospitalized for asthma in past year

23. How many times (have you/has your child) gone to a hospital emergency room, or walk-in clinic (CA)/gone to an accident and casualty room/been visited by an emergency mobile doctor or gone to an emergency room (GER)/gone to an emergency room, or been visited by a night-shift doctor (ITA)/gone to an emergency room (SPN)/gone to an accident and casualty room or after-hours service (UK) in the past 12 months?

24. How many times (have you/has your child) had an urgent care visit to these places for asthma in the past 12 months?

25. How many times (have you/has your child) gone to see a doctor or other health provider in the past 12 months about asthma exacerbations or worsening symptoms?

26. (Have you/Has your child) EVER had an asthma episode so bad that (you/your child):
    • Had to stop exercising?
    • Had to leave work/school?
    • Had to be put in the ICU of a hospital?

27. (Have you/Has your child) ever had an asthma episode so bad that you thought (your/your child's) life was in danger?

28. When was the most recent time (you/your child) had an asthma episode so bad that you thought (your/your child's) life was in danger: within the past month, within the past 6 months, within the past year, more than a year ago, or never?

Asthma burden

29. Has (your/your child's) asthma caused (you/your child) to miss work or school in the past year?

30. How many work or school days (have you/has your child) lost in the past year as a result of asthma?

31. Thinking about productivity on a scale of 0 to 100, where 100 means 100% productivity, where would you rank (your/your child's) productivity on a typical day?

32. Where would you rank (your/your child's) productivity on the same scale of 0 to 100, at times of the year when (your/your child's) asthma/health was at its worst?

33. How much do you feel that (your/your child's) asthma limits what (you/your child) can do in each of the following areas: a lot, some, only a little or not at all?

34. As a result of (your/your child's) asthma, how often (do you/does he or she) feel:
    • Tired or fatigued?
    • Fearful?
    • Depressed or blue?
    • Embarrassed?
    • Frustrated?
    often, sometimes, rarely, or never?

35. Which has a greater impact on (your/your child's) quality of life — day-to-day asthma symptoms or sudden severe asthma episodes, or are they about the same?

    Base: Had sudden severe episode in past year

Asthma management

36. How does (your doctor/your child's doctor) usually assess (your/your child's) asthma? Does he or she usually:
    • Have you fill out a questionnaire?
    • Give you a breathing test or spirometry?

37. A lung function test measures how much and how quickly you can move air out of your lungs. For this test, you breathe into a mouthpiece attached to a recording device. Has (your/your child's) doctor ever given (you/your child) a lung function test?

38. (Do you/Does your child) have a peak flow meter?

39. Has (your/your child's) doctor or practice nurse developed a written action plan for (your/your child's) asthma treatment?

40. Did the doctor or health professional:
    - Give you a breathing test?
    - Have you fill out a questionnaire?
    - Order a chest X-ray?

    Base: Saw doctor due to asthma exacerbations

41. Did the doctor or health professional:
    - Prescribe a new controller medicine OR tell you to take your controller more often?
    - Prescribe a new rescue medicine OR tell you to take rescue medicine more often?
    - Prescribe an oral steroid?

    Base: Saw doctor due to asthma exacerbations

Asthma medication and treatment

42. In the past 4 weeks, (have you/has your child) used any prescription medicine [such as blue puffer (CAN), such as blue inhaler (UK)] to give (you/your child) relief or rescue from asthma symptoms?

43. How often (do you/does your child) use an inhaler for relief or rescue from asthma symptoms: daily, 3-6 times a week, 1-2 times a week, or less than once a week?

44. How many inhalers of the relief medicine for (your/your child's) asthma (do you/does he/she) use per year?

    Base: Used inhaler for quick-relief medicine

45. In the past 4 weeks, (have you/has your child) used any prescription medicine for control, prevention, maintenance, or regular treatment (CAN)/long-term maintenance or continuous treatment (GER)/control or maintenance (ITA)/maintenance treatment (SPN)/long-term control or prevention (UK) of (your/his/her) asthma?

46. How often (do you/does your child) take this medicine: every day at least twice a day, every day once a day, 4 to 6 days a week, 2 to 3 days a week, once a week, 2 to 3 times a month, once a month, less often than once a month, or as needed for symptoms?

47. For how long did (you/your child) stop taking (your/your child's) asthma control or prevention medicine the last time: a few days, 1 to 2 weeks, 3 to 4 weeks, 1 to 2 months, 3 to 5 months, or 6 months or longer?

    Base: Took controller medicine in past year

48. (Have you/Has your child) had to take an oral steroid (pill or liquid) to manage (your/your child's) asthma symptoms in the past 12 months?

49. In the past 4 weeks, (have you/has your child) used any over-the-counter medicine to give (you/your child) relief from asthma symptoms?

50. In the past 4 weeks, (have you/has your child) used any prescription medicine [such as blue puffer (CAN), such as blue inhaler (UK)] to give (you/your child) relief or rescue from asthma symptoms?

51. In the past 4 weeks, (have you/has your child) used any prescription medicine for control, prevention, maintenance, or regular treatment (CAN)/long-term maintenance or continuous treatment (GER)/control or maintenance (ITA)/maintenance treatment (SPN)/long-term control or prevention (UK) of (your/his/her) asthma?

52. When was the most recent time that (you/your child) took an oral steroid to manage (your/your child's) asthma symptoms: within the past week, within the past 4 weeks, within the past 6 months, or within the past year?

Attitudes about asthma

53. Now I would like you to tell me how much you agree or disagree with the following statements. Do you strongly agree, somewhat agree, neither agree nor disagree, somewhat disagree, or strongly disagree that:
    - Maintenance medicines should be taken every day?
    - Maintenance medicines are not necessary when asthma symptoms are not experienced regularly?
    - Rescue medicines can be used every day if needed?
    - Fear of asthma exacerbations keeps me from doing the things I want to?
    - I worry about using oral steroids, like prednisone?

54. Would you consider (your/your child's) asthma well controlled if…
    - You have only 2 urgent doctor visits for asthma per year?
    - You have only 1 emergency room visit for asthma per year?
    - Your asthma bothers you less than half the time when you exercise?
    - You have to take quick-relief medicine only 3 times a week?
    - You have exacerbations only 3 or 4 times a year?

CA, Canada, **GER**, Germany, **ITL**, Italy, **SPN**, Spain, **UK**, United Kingdom.
